# Application of a Novel ‘Make and Test in Parallel’ Strategy to Investigate the Effect of Formulation on the Pharmacokinetics of GDC-0810 in Healthy Subjects

**DOI:** 10.1007/s11095-018-2516-0

**Published:** 2018-10-15

**Authors:** Sravanthi Cheeti, Hao Helen Hou, Eric Nelson, Helen Walker, Buyun Chen, Roland Morley, Mary Gates, Luna Musib, Sandhya Girish, Srikumar Sahasranaman, Lichuan Liu

**Affiliations:** 1 Genentech, Inc., 1 DNA Way, MS# 463A, Genentech, South San Francisco, California 94080 USA; 2 Quotient Sciences, Mere Way, Ruddington, Nottingham, UK

**Keywords:** food effect, formulation optimization, GDC-0810, pharmacokinetics, relative bioavailability

## Abstract

**Purpose:**

GDC-0810, administered orally, was used in Phase I and II clinical studies to treat estrogen receptor positive breast cancers. It contains N-methyl-D-glucamine (NMG) salt of GDC-0810 with 10% sodium lauryl sulfate (SLS) as a surfactant and 15% sodium bicarbonate (NaHCO_3_) as an alkalizing agent to aid dissolution. To improve the processability of the formulation and reduce potential mucosal irritation in future Phase III clinical studies, the salt form and the amount of excipient required further optimization. To achieve this, we employed a novel “Make and Test in Parallel” strategy that facilitated selecting formulation in a rapid timeframe.

**Methods:**

RapidFACT®, a streamlined, data-driven drug product optimization platform was used to bridge Phase I/II and Phase III formulations of GDC-0810. Five prototype formulations, varying in either the form of active pharmaceutical ingredient and/or the levels of the excipients SLS and NaHCO_3_ were assessed. Uniquely, the specific compositions of formulations manufactured and dosed were selected in real-time from emerging clinical data.

**Results:**

The study successfully identified a Phase III formulation with a reduced SLS content, which when administered following a low-fat meal, gave comparable pharmacokinetic exposure to the Phase I/II formulation administered under the same conditions.

**Conclusions:**

Our novel ‘Make and Test in Parallel’ approach enabled optimization of GDC-0810 formulation in a time- and cost-efficient fashion.

## Introduction

Breast cancer is the most common form of cancer and the leading cause of cancer death in women worldwide, accounting for more than 1,670,000 new cases and nearly 522,000 cancer deaths annually (1). Approximately 70% of all breast cancers express and are dependent on the estrogen receptor (ER) for tumor growth and progression (2). Modulation of estrogen activity and/or synthesis is the mainstay of therapeutic approach in postmenopausal women with ER-positive (ER^+^) breast cancer. Despite the effectiveness of available hormonal therapies such as tamoxifen, aromatase inhibitors, and ER antagonists/degraders (3), many patients relapse or develop resistance to these agents and therefore require further treatment for optimal disease control, thus representing a population with an unmet medical need. As such, there is a need for new ER-targeting therapies with increased anti-tumor activity to further delay disease progression and/or overcome resistance to the currently available hormonal therapies and ultimately prolong survival in women with ER^+^ advanced breast cancer.

GDC-0810 was developed as an oral drug for use as a single agent, or in combination, for treatment of estrogen receptor positive (ER^+^), human epidermal growth factor receptor 2 negative (HER2^−^) breast cancer (4). The molecule, GDC-0810, was however a weak acid with low intrinsic solubility (< 0.06 μg/ml), an acidic pK_a_ of 4.3 and a log *P* value of 6.2. GDC-0810 N-Methyl-D-glucamine (NMG) salt was selected as the solid form for clinical development as an immediate release tablet formulation using a dose strength of 200 mg (free acid equivalent) for use in Phase I and II clinical studies at a single daily dose of 600 mg. In general, the salt form of a weakly ionizable acid or base is expected to have a higher dissolution rate than the free form. However, due to super-saturation, the salt may sometimes precipitate out as the insoluble free form upon dissolution, forming a layer on the surface of the dissolving salt, which may result in low bioavailability of the drug. The addition of functional excipients, such as surfactants and pH modifiers, in the dosage forms have been reported to be an effective tool in improving the dissolution behavior of compounds with pH dependent solubility (5,6). Further, *in vitro* dissolution studies have shown that incorporating 15% sodium bicarbonate (NaHCO_3_) as an alkalizing agent in the solid dosage form of GDC-0810 can enhance its release via micro-environmental pH modulation, resulting in increased dissolution (7).

In addition to 15% NaHCO_3_, the Phase I/II formulation also contained 10% sodium lauryl sulfate (SLS) as a surfactant. As the percentage of SLS, a mucosal irritant, used in this formulation was rather high, some gastro-intestinal toxicities were expected (8). Additionally, the high level of SLS resulted in poor processability of the formulation and caused significant challenges during tablet manufacturing. Moreover, GDC-0810 N-Methyl-D-glucamine (NMG - 195 g/mol) salt also added substantial counter ion weight to the formulation, thus decreasing the formulation space for a high drug-loading tablet. In summary, the amount of excipient in the formulation that was being used in the Phase I/II studies appeared to be suboptimal given early clinical data indicating a potential risk of diarrhea with GDC-0810. Therefore, further optimization of this formulation was required for clinical studies.

The ‘conventional’ process for development of formulations relies on pre-clinical models to screen prototype formulations and select candidates for clinical evaluation. These candidates are then scaled up and characterized to provide a package of CMC data supporting a product shelf life of at least three months, prior to proceeding to a human clinical study to establish whether the target product profile (TPP) has been met. The only formulation(s) available for dosing in the relative bioavailability study are therefore manufactured weeks or months in advance. However, the poor predictive power of pre-clinical models as previously reported (9) is a limitation and the TPP is frequently not achieved in the first iteration, which often requires multiple cycles of *in vitro*, pre-clinical, product manufacturing and clinical dosing to optimize the formulation, significantly increasing the time and cost of development.

To address this limitation, we employed a novel ‘Make and Test in Parallel’ strategy for rapid development and optimization using Rapid Formulation development and Clinical Testing (RapidFACT®). RapidFACT® is a streamlined, data-driven approach for formulation optimization under which drug products can be manufactured within 7 days of dosing at a small scale, removing scale-up and stability package generation from the critical path to obtaining clinical data on product performance. In addition, the integrated GMP manufacturing and clinical research facility allows clinical data from one study period to be reviewed and used to select the product to be manufactured and evaluated on the next dosing occasion, with typically only 14 days between dosing occasions. This therefore allows a series of formulations to be iteratively ‘made and tested’ as part of a crossover study design within a single group of volunteers. This approach has been successfully applied to optimize the dose and bioavailability of drugs in over 200 formulations (10–14). This strategy uses real-time adaptive manufacturing to support an adaptive study design. Decision-making data are generated using the proposed study design to inform formulation selection in a rapid timeframe. This provides flexibility in adjusting formulation composition based on the rapid generation and analysis of interim clinical data results in reduced consumption of Active Pharmaceutical Ingredient (API), accelerated timelines, and overall cost-effective decision-making. RapidFACT® flexibility can be maximized through the definition of a ‘formulation design space’, bracketing levels of critical-to-performance components in the formulation from which compositions can be selected during clinical conduct. By generating representative technical data prior to the study at the ‘corner point’ compositions of the design space, assurance of product quality of any specific compositions described therein is provided (11).

A relative bioavailability study, using the above-mentioned approach, was conducted to identify a prototype formulation with the optimal form of the active pharmaceutical ingredient as well as the optimal concentration of SLS and/or NaHCO_3_, which would provide comparable pharmacokinetics to the Phase I/II formulation. By using the ‘Make and Test in Parallel’ approach, a total of five prototype formulations of GDC-0810, varying in either the form of active pharmaceutical ingredient and/or the levels of the excipients SLS and NaHCO_3_ (2–10% and 5–15%, respectively), were studied. In addition, the effect of food on the bioavailability of the selected formulation was investigated.

## Materials and Methods

This study was conducted at Quotient Sciences (Nottingham, UK), in full conformance with the ICH E6 guideline for GCP and the principles of the Declaration of Helsinki, or the laws and regulations of the country in which the research is conducted, whichever afforded the greater protection to the individual. The study complied with the requirements of the ICH E2A guideline (Clinical Safety Data Management: Definitions and Standards for Expedited Reporting) and the E.U. Clinical Trial Directive (2001/20/EC). All subjects provided written informed consent.

The study was conducted as a RapidFACT® program which uses real-time adaptive manufacturing and screening of new formulations in humans using Quotient’s integrated GMP manufacturing and clinical research facility. Using this platform, drug products were selected within a continuous formulation design space where a range of product performance attributes was obtained by varying the quantitative composition of SLS and NaHCO_3_ in the formulation.

### Tablets

**Fig. 1:**
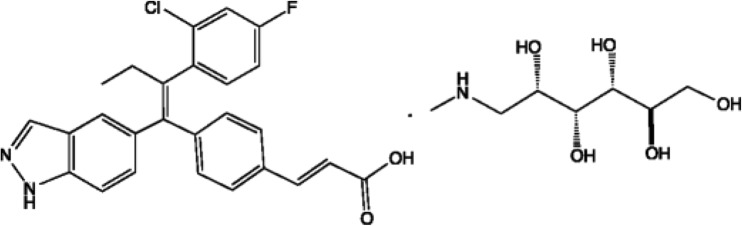
Molecular Structure of GDC-0810 NMG Salt

Reference GDC-0810 Phase II NMG salt tablets were supplied by Genentech Inc. as 200 mg tablets for oral administration packaged as per the product label. The molecular structure of GDC-0810 NMG salt is shown in Fig. 1. The GDC-0810 drug substance and drug product intermediate for the manufacture of the Phase III prototype tablets were also provided by Genentech Inc. GDC-0810 Phase III prototype tablets were manufactured and supplied in real-time by Quotient Sciences as 200 mg tablets for oral administration. The composition of Phase II and III GDC-0810 tablets administered in the relative bioavailability study are summarized in Table I

**Table I:** Composition of Phase II and III GDC-0810 Tablets Administered in Parts 1, 2, and 3 of the Relative Bioavailability Study

IMP	Composition			Treatment
	API form	SLS	NaHCO_3_	
Phase II Tablet	NMG salt	10%	15%	A^a^, E^a^ and H^a^
Phase III Prototype Tablet Formulations				
Prototype 1	NMG salt	2%	15%	B^a^
Prototype 2	NMG salt	2%	10%	C^a^
Prototype 3	NMG salt	2%	5%	D^a^
Prototype 4	Free acid	2%	15%	F^a^
Prototype 5	Spray-dried amorphous solid dispersion	2%	15%	G^a^
Prototype 1	NMG salt	2%	15%	I^b^
Prototype 1	NMG salt	2%	15%	J^a^

*IMP*, investigational medicinal product; *API*, active pharmaceutical ingredient; *NMG*, N-methyl glucamine; *NaHCO*_*3*_, sodium bicarbonate; *SLS*, sodium lauryl sulfate

^a^Administered 30 min after the start of a low-fat meal

^b^Administered in the fasted state (minimum 8-h fast)

### Clinical Study Design

This relative bioavailability study was a Phase I, single center, open-label, randomized, 3-part crossover study in separate cohorts of healthy female subjects of non-childbearing potential. Each study part consisted of a screening period of up to 28 days, a study period of 4 days (Day −1 to Day 3; 4 periods in Part 1 and 3 periods in each of Parts 2 and 3) in which study drug was administered on Day 1 of each period, and a minimum follow-up period of 28 days. Study participation for each subject was approximately 10 weeks.

Blood samples for the measurement of GDC-0810 plasma concentrations were obtained through 48 h post dose in each period. There was a minimum washout period of 3 days between each dose administration based on the mean half-life of approximately 8 h in patients. Safety data, in the form of repeated measures of vital signs, electrocardiograms (ECGs), clinical examinations, clinical laboratory testing, and a record of adverse events (AEs), were recorded.

The primary objective of Part 1 of the study was to determine the relative bioavailability of GDC-0810 Phase III prototype formulations, with varying levels of the excipients, SLS and NaHCO_3_, with respect to the control (Phase II tablet formulation). The primary objective of Part 2 of the study was to determine the relative bioavailability of GDC-0810 Phase III prototype formulations, with varied form of active pharmaceutical ingredient i.e., Free Acid and Spray-Dried Amorphous Dispersion (and optimal levels of excipients identified in Part 1), with respect to the control (Phase II tablet formulation). Subjects in both Parts 1 and 2 received 200 mg dose with a standardized low-fat food to mimic recommended prandial condition in the clinical studies. The primary objective of Part 3 was to assess the PK of GDC-0810 using a Phase III prototype tablet formulation selected from Parts 1 and 2 (administered with low-fat food or under fasted conditions) at the current clinical dose of 600 mg with respect to the current Phase II tablet formulation administered with low fat food.

Healthy female volunteers of non-childbearing potential were chosen for this study to assess the PK effects of GDC-0810 within a dose range that was expected to be well tolerated. No formal sample size calculation was performed. However, a minimum sample size of 12 healthy volunteers for each part of the study was considered sufficient to achieve the objectives.

#### Parts 1 & 2

**Fig. 2:**
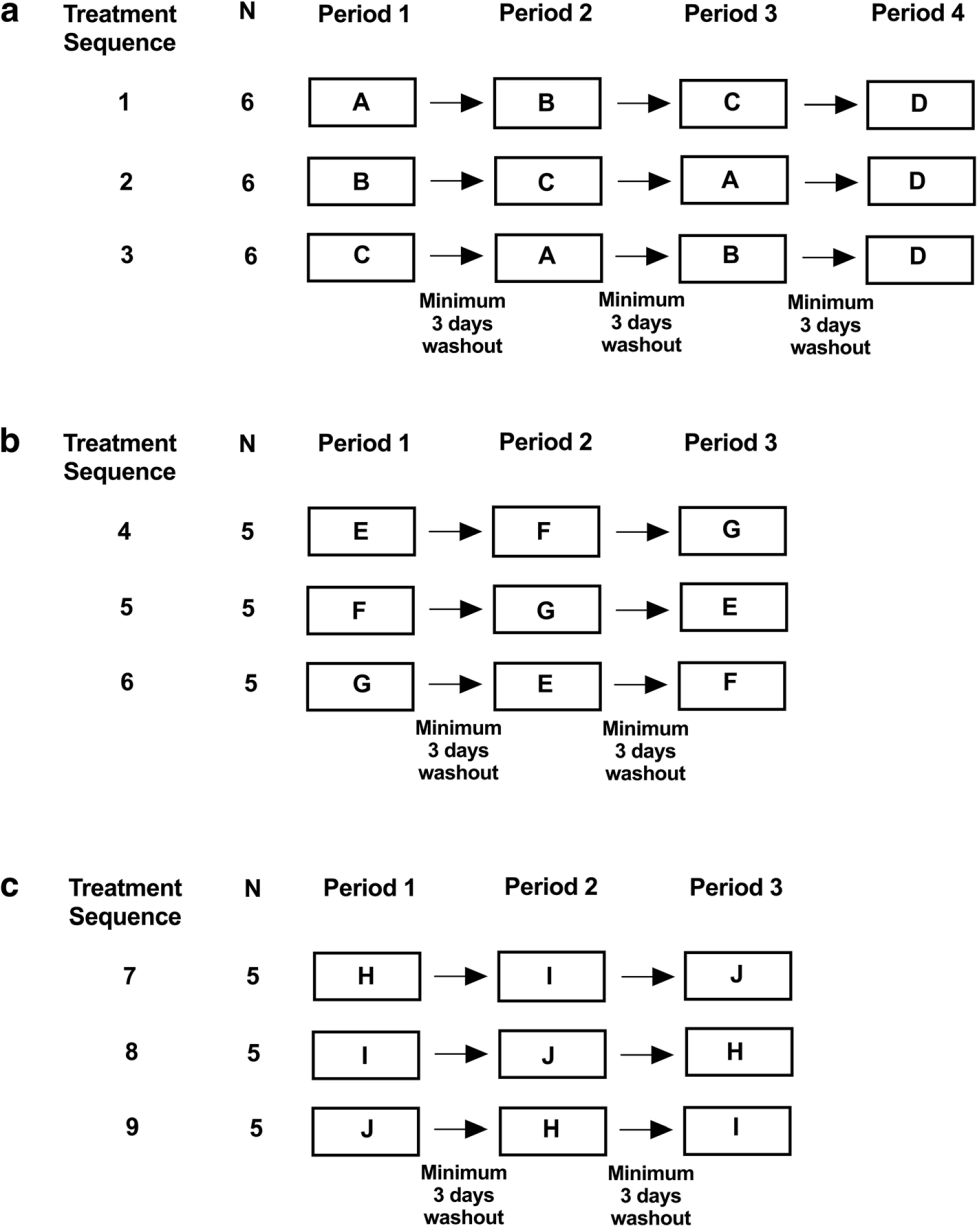
**Study Schematic for Parts 1, 2, and 3 of the Relative Bioavailability Study.** **Panel** (**a**)**:** Treatment A: 200 mg Phase II tablet; NMG salt; 10% SLS and 15% NaHCO3. Treatment B: 200 mg Phase III Prototype 1 tablet; NMG salt; 2% SLS and 15% NaHCO3. Treatment C: 200 mg Phase III Prototype 2 tablet; NMG salt; 2% SLS and 10% NaHCO3. Treatment D: 200 mg Phase III Prototype 3 tablet; NMG salt; 2% SLS and 5% NaHCO3 (formulation parameters were determined following Period 3). **Panel** (**b**)**:** Treatment E: 200 mg Phase II tablet; NMG salt; 10% SLS and 15% NaHCO3. Treatment F: 200 mg Phase III Prototype 4 tablet; Free acid; 2% SLS and 15% NaHCO3. Treatment G: 200 mg Phase III Prototype 5 tablet; Spray-dried amorphous solid dispersion; 2% SLS and 15% NaHCO3 **Panel** (**c**)**:** Treatment H: 3 × 200 mg Phase II tablet (NMG salt; 10% SLS and 15% NaHCO3), administered 30 min after a low-fat meal. Treatment I: 3 × 200 mg Phase III Prototype tablet selected from Parts 1 & 2 (NMG salt; 2% SLS and 15% NaHCO3), administered in the fasted state. Treatment J: 3 × 200 mg Phase III Prototype tablet selected from Parts 1 & 2 (NMG salt; 2% SLS and 15% NaHCO3), administered 30 min after a low-fat meal.

Part 1 was a four-period crossover design to investigate the effect of excipient levels on the PK of GDC-0810 administered (Fig. 2a). A total of 17 subjects were enrolled in Part 1 to complete a minimum of 12 subjects in each study period. Part 2 was a 3-period crossover design to investigate the effect of varied form of active pharmaceutical ingredient on the PK of GDC-0810 administered (Fig. 2b). A total of 15 subjects were enrolled in Part 2 to complete a minimum of 12 subjects in each study period.

On Day 1 of each study period, each subject received a single 200 mg dose of GDC-0810 (as a Phase II tablet or one of five Phase III prototype tablet formulations) administered 30 min after the start of the low-fat meal across all periods. Subjects were randomized to one of the treatment sequences for Treatments A, B, C in Part 1 and Treatments E, F, G in Part 2, with an additional fixed treatment consisting of Treatment D in Part 1. An interim decision was made, based on the available safety, tolerability, and PK data following completion of Period 3, to select the formulation to be dosed in Period 4 in Part 1. The decision to proceed with Part 2 was taken after review of the PK, safety, and tolerability data from Part 1 of the study. Following review of safety, tolerability, and PK data from Parts 1 and 2, the decision to proceed with Part 3 was taken.

#### Part 3

Part 3 was a 3-period crossover design to compare the PK of a Phase III prototype tablet formulation, selected from Parts 1 and 2, with the Phase II tablet formulation (both administered after ingestion of a low-fat meal) at the current clinical dose of 600 mg, and to investigate the PK of the selected Phase III prototype formulation in the fasted state (Fig. 2c). A total of 15 subjects were enrolled in Part 3 to complete a minimum of 12 subjects in each study period.

On Day 1 of each study period, each subject received a single dose of 600 mg GDC-0810 administered as 3 × 200 mg across 3 periods; a) as a Phase II tablet administered 30 min after the start of a low-fat meal, b) as a Phase III prototype tablet formulation administered in the fasted state, and c) as a Phase III prototype tablet formulation administered 30 min after the start of a low-fat meal. The composition of Phase II tablet and Phase III prototype tablet used in Part 3 was based on the review of available data from Parts 1 and 2. Subjects were randomized to one of three treatment sequences.

### Dose Rationale

The recommended clinical dose of GDC-0810 was 600 mg, administered orally as three 200 mg tablets. It was demonstrated in a clinical Phase I/IIa study that increasing the dose of GDC-0810 over the range of 100 mg to 800 mg resulted in a dose proportional increase in plasma exposure. Based upon the clinical experience with GDC-0810 to date, GDC-0810 was shown to be well tolerated in patients with late stage breast cancer up to repeat daily doses of 600 mg. Therefore, 200 mg single dose was selected for Parts 1 and 2 of this study in healthy volunteers to ensure that there was sufficient safety margin to test the new formulations while staying within the linear dose range. Upon review of PK, safety and tolerability of the optimal Phase III prototype formulation identified in Parts 1 and 2, Part 3 of the study was conducted at the clinically relevant dose of 600 mg in fasted and fed conditions.

### Pharmacokinetic Assessments

Venous blood samples were collected for the analysis of plasma concentrations and PK parameter estimations of GDC-0810 at pre-dose, 0.5, 1, 2, 3, 4, 6, 8, 12, 16, 24, 36, and 48 h post dose in each part. Plasma concentrations of GDC-0810 were determined using validated bioanalytical methods (Covance Laboratories Ltd., Harrogate, UK). The lower limit of quantification (LLOQ) was 5 ng/ml.

Pharmacokinetic parameter estimates of plasma concentrations of GDC-0810 for each study part were obtained using Phoenix WinNonlin PK software (v6.3, Certara USA, Inc., St. Louis, MO, USA).

### Statistical Analysis

In Parts 1 and 2, the effect of formulation on the PK of GDC-0810 was evaluated. In Part 3, the effect of food on the PK of the selected Phase III prototype formulation was evaluated, and a comparison of the selected Phase III prototype tablet with the Phase II tablet after ingestion of a low-fat meal was made.

Phase III prototype tablets 1, 2, or 3 were compared against Phase II tablet in Part 1 and Phase III prototype tablets 4 or 5 were compared against Phase II tablet in Part 2. All the tablets in Parts 1 & 2 were administered after ingestion of a low-fat meal.

Phase III prototype tablet selected from Parts 1 and 2 was also compared against Phase II tablet, both administered after ingestion of a low-fat meal, in Part 3. In addition, the effect of low-fat food *vs*. fasted conditions on Phase III prototype tablet was compared in Part 3.

Formal statistical analyses for all treatments were performed on the PK parameters C_max_, AUC_0-t_, and AUC_0-inf_. The PK parameters underwent natural logarithmic transformations and were subsequently analyzed using mixed effect analysis of variance (ANOVA) modeling techniques. The model included terms for treatment (i.e., GDC-0810 tablet, by formulation), period (Part 2 only) and sequence as fixed effects, and subject nested within sequence as a random effect. Adjusted geometric mean ratios (GMRs) and 90% confidence intervals (CIs) for the adjusted GMRs for the pairwise treatment comparisons were calculated, where the ratio was defined as test/reference or fed/fasted. *P*-values for the null hypothesis that the GMR = 100% were presented. All decisions concerning the formulation selection for GDC-0134 were based on these *p*-values estimated based on relative exposures with respect to the reference.

## Results

### Demographic, Safety, and Tolerability Data

Seventeen healthy female subjects (16 white and 1 Asian) between 50 and 69 years of age were enrolled into Part 1 of the study. Fifteen healthy female subjects (14 white and 1 Asian) between 50 and 69 years of age were enrolled into Part 2 of the study. Thirteen healthy female subjects (11 white and 2 Asian) between 46 and 68 years of age were enrolled into Part 3 of the study.

Fifteen out of the 17 enrolled subjects in Part 1 completed the study in accordance with the protocol. Oral administration of GDC-0810 at single dose levels of 200 mg and 600 mg was generally well tolerated under the conditions of the study. There were no serious adverse event (SAEs), severe or life-threatening treatment emergent adverse events (TEAEs, CTCAE Grade 3 or more), or TEAEs leading to death. There were no clinically significant changes in vital signs, electrocardiographs (ECGs), or physical examination findings recorded during the study.

### Pharmacokinetic Results

#### Part 1

In Part 1, three prototype formulations of GDC-0810 as NMG salt (200 mg tablets) were studied (Treatments B, C and D); each prototype varied in the levels of the excipients SLS and NaHCO_3_. Treatment A (Phase II tablet) had 10% SLS and 15% NaHCO_3_; Treatment B (Phase III prototype 1) had 2% SLS and 15% NaHCO_3_; Treatment C (Phase III prototype 2) had 2% SLS and 10% NaHCO_3_; and Treatment D (Phase III prototype 3) had 2% SLS and 5% NaHCO_3_.

**Fig. 3:**
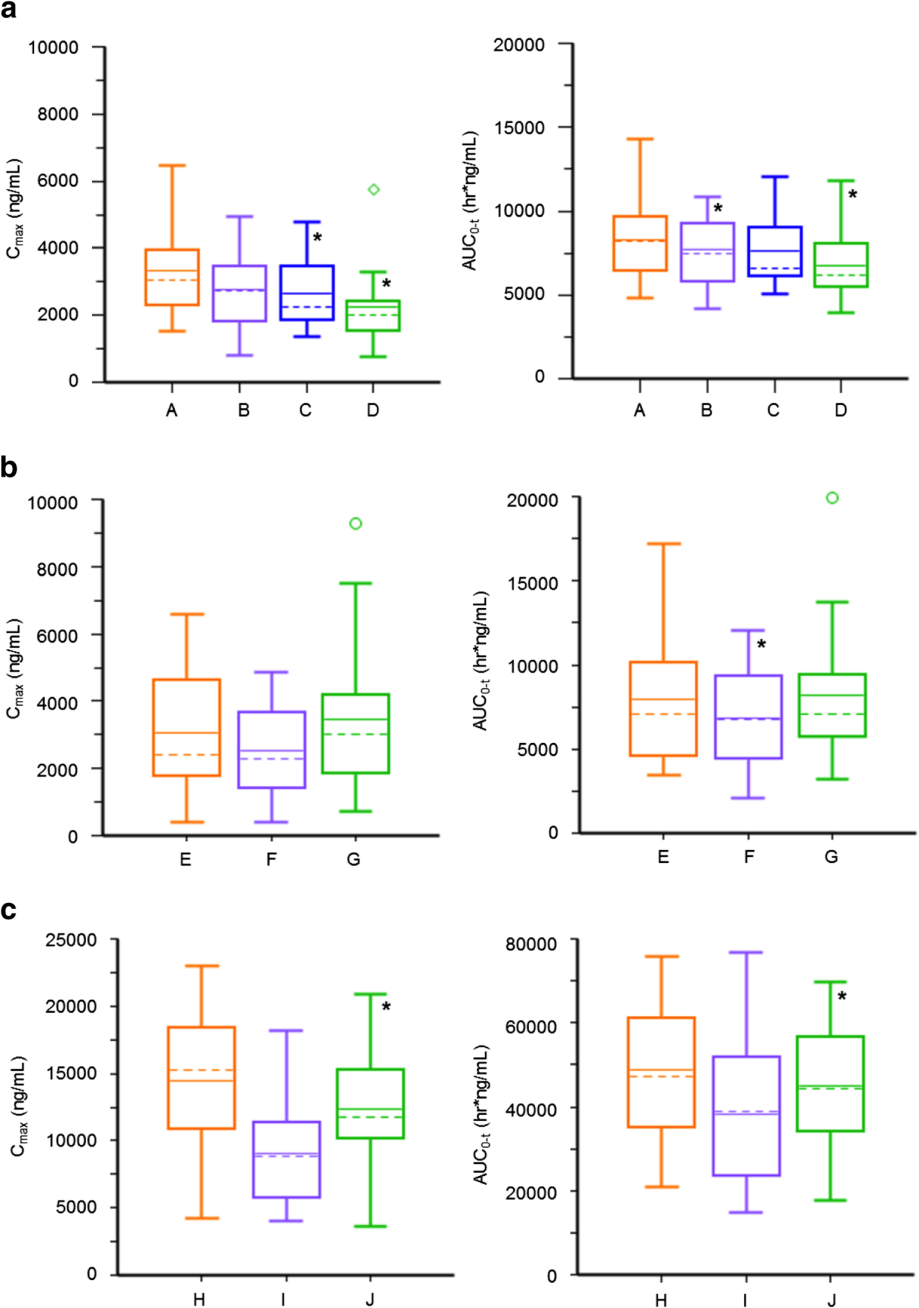
**Comparison of GDC-0810 Plasma PK.** **Panel** (**a**)**:** Treatments A to D in Part 1 with 200 mg GDC-0810. **Panel** (**b**)**:** Treatments E to G in Part 2 with 200 mg GDC-0810; and **Panel** (**c**)**:** Treatments H to J in Part 3 with 600 mg GDC-0810. Dotted line represents median; Solid line represents arithmetic mean; box represents interquartile range (IQR; 25th–75th quartiles); IQR tails default to 1.5xIQR and values beyond IQR tails shown as outliers; * indicates statistically significant difference relative to the reference.

Plasma concentration-time profiles and PK parameters of GDC-0810 following administration of Treatments A to D are shown in Fig. 3a and Table II, respectively. Geometric mean ratios (GMR) of GDC-0810 as Treatments B, C, and D relative to the reference formulation (Treatment A) are summarized in Table II.

**Table II:** Geometric Mean (Geometric CV%) Pharmacokinetic Parameters for GDC-0810 Following a Single Oral Dose of 200 mg GDC-0810 (Treatments A to D): Part 1

	Treatment^a^			
PK Parameter	A 200 mg GDC-0810 Phase II Tablet NMG salt; 10% SLS, 15% NaHCO_3_ (*n* = 17)	B 200 mg GDC-0810 Phase III Prototype 1 Tablet NMG salt; 2% SLS, 15% NaHCO_3_ (n = 15)	C 200 mg GDC-0810 Phase III Prototype 2 Tablet NMG salt; 2% SLS, 10% NaHCO_3_ (n = 17)	D 200 mg GDC-0810 Phase III Prototype 3 Tablet NMG salt; 2% SLS,5% NaHCO_3_ (n = 15)
t_max_^b^ (h)	2.00 (1.00–4.00)	3.00 (1.00–4.02)	3.00 (1.00–4.00)	2.00 (0.50–4.00)
C_max_ (ng/ml)	3140 (37.2)	2540 (47.4)	2440 (42.8)	2010 (49.5)
AUC_0-t_ (hr*ng/ml)	8020 (28.7)	7420 (29.0)	7310 (26.2)	6460 (30.5)
t_1/2_ (h)	18.14 (73.7) [*n* = 13]	17.41 (48.0) [*n* = 12]	14.33 (36.1) [*n* = 12]	13.40 (77.5) [*n* = 11]
GMR (90% CI) of C_max_ (%)	NA	82% (66–102%)	78% (63–96%)	66% (53–82%)
GMR (90% CI) of AUC_0-t_ (%)	NA	90% (82–100%)	91% (83–100%)	83% (75–91%)

*NA*, not applicable

^a^All treatments were administered 30 min after the start of a low-fat meal

^b^Median (range)

Treatments C and D showed statistically significantly lower levels of peak exposure, as measured by C_max_ (approximately 22% lower; *p* = 0.05 and 34% lower; *p* = 0.002, respectively), when compared to the reference. However, there was no significant difference in peak exposure between Treatment B and reference (*p* = 0.12).

In addition, Treatments B and D showed statistically significantly lower levels of total exposure, as measured by AUC_0–t_ when compared to Treatment A reference formulation (10% lower; *p* = 0.085 and 18% lower; p = 0.002, respectively). However, there was no significant difference in total exposure, when measured by AUC_0–t_, between Treatment C and reference (*p* = 0.11).

#### Part 2

In Part 2, two further prototype formulations of GDC-0810 as 200 mg tablets were studied; each prototype varied in the form of active pharmaceutical ingredient. Treatment E (Phase II tablet) had NMG salt, 10% SLS and 15% NaHCO_3_; Treatment F (Phase III prototype 4) was a Free Acid, with 2% SLS and 15% NaHCO_3_; and Treatment G (Phase III prototype 5) was a Spray-Dried Amorphous Dispersion, with 2% SLS and 15% NaHCO_3_.

Plasma concentration-time profiles and PK parameters of GDC-0810 following administration of Treatments E to G are shown in Fig. 3b and Table III, respectively. GMRs of GDC-0810 as Treatments F and G relative to the reference formulation (Treatment E) are summarized in Table III.

**Table III:** Geometric Mean (Geometric CV%) Pharmacokinetic Parameters for GDC-0810 Following a Single Oral Dose of 200 mg GDC-0810 (Treatments E to G): Part 2

	Treatment^a^		
PK Parameter	E 200 mg GDC-0810 Phase II Tablet NMG salt; 10% SLS, 15% NaHCO_3_(*n* = 15)	F 200 mg GDC-0810 Phase III Prototype 4 Tablet Free Acid; 2% SLS, 15% NaHCO_3_ (n = 15)	G 200 mg GDC-0810 Phase III Prototype 5 Tablet Spray-Dried Amorphous Dispersion; 2% SLS, 15% NaHCO_3_ (n = 15)
t_max_^b^ (h)	2.00 (1.00–4.00)	3.10 (2.00–4.07)	3.05 (1.00–4.02)
C_max_ (ng/ml)	2510 (80.4)	2070 (85.8)	2830 (75.5)
AUC_0-t_(hr*ng/ml)	7180 (50.2)	6180 (53.0)	7330 (51.9)
t_1/2_ (h)	14.56 (38.5) [*n* = 9]	18.57 (46.2) [n = 9]	18.53 (34.3) [n = 12]
GMR (90% CI) of C_max_ (%)	NA	82% (67–102%)	113% (91–139%)
GMR (90% CI) of AUC_0-t_ (%)	NA	86% (79–94%)	102% (94–111%)

*NA*, not applicable

^a^All treatments were administered 30 min after the start of a low-fat meal

^b^Median (range)

There was no significant difference in peak exposure, as measured by C_max_, between each of Treatments F and G when compared to the reference (*p* = 0.13 and *p* = 0.35, respectively). There was also no statistically significant difference in total exposure between Treatment G compared to reference when measured by AUC_0-t_ (*p* = 0.69). However, Treatment F showed statistically significantly lower levels of total exposure compared to Treatment E reference formulation when measured by AUC_0–t_ (14% lower; *p* = 0.006).

#### Part 3

In Part 3, the Phase III prototype formulation (selected from Part 2) of GDC-0810 as 600 mg tablets was studied with low-fat food and under fasted conditions along with the Phase II tablet (reference) administered with low-fat food. The dose for all treatments was 600 mg. Treatment H was Phase II tablet administered with low-fat meal; Treatment I was Phase III Prototype 1 tablet administered under fasted conditions; and Treatment J was Phase III Prototype 1 tablet administered with low-fat meal.

Plasma concentration-time profiles and PK parameters of GDC-0810 following administration of a single oral dose of 600 mg GDC-0810 as Treatments H to J are shown in Fig. 3c and Table IV, respectively. GMRs of GDC-0810 as Treatments I and J relative to the reference formulation (Treatment H) are summarized in Table IV.

**Table IV:** Geometric Mean (Geometric CV%) Pharmacokinetic Parameters for GDC-0810 Following a Single Oral Dose of 600 mg GDC-0810 (Treatments H to J): Part 3

	Treatment		
PK Parameter	H 600 mg GDC-0810 Phase II Tablet NMG salt; 10% SLS, 15% NaHCO_3_ Fed^a^ (*n* = 13)	I 600 mg GDC-0810 Phase III Prototype 1 Tablet NMG salt; 2% SLS, 15% NaHCO_3_ Fasted^a^ (n = 13)	J 600 mg GDC-0810 Phase III Prototype 1 Tablet NMG salt; 2% SLS, 15% NaHCO_3_ Fed^a^ (n = 13)
t_max_^b^ (h)	3.00 (2.00–4.00)	1.00 (0.93–4.00)	3.00 (2.00–4.02)
C_max_ (ng/ml)	13,000 (57.0)	8320 (46.4)	11,200 (54.4)
AUC_0-t_ (hr*ng/ml)	48,100 (41.1)	35,900 (49.8)	44,700 (38.5)
t_1/2_ (h)	14.82 (37.5)[*n* = 10]	14.15 (50.7)[n = 9]	15.87 (46.4)[n = 10]
GMR (90% CI) of C_max_ (Fed *vs* Fast) (%)	NA	NA	137% (109–171%)
GMR (90% CI) of AUC_0-t_ (Fed *vs* Fast) (%)	NA	NA	126% (107–148%)
GMR (90% CI) of C_max_ (Test *vs* Ref) (%)	NA	NA	88% (70–110%)
GMR (90% CI) of AUC_0-t_ (Test *vs* Ref) (%)	NA	NA	94% (80–111%)

*NA*, not applicable

^a^Fed: treatment administered 30 min after the start of a low-fat meal; fasted: treatment administered after a minimum 8-h fast

Fed *vs*. fasted: Treatment J *vs*. Treatment I, Test *vs*. Ref; Treatment J *vs*. Treatment H

^b^Median (range)

Treatment J showed significantly higher levels of total exposure when administered in the fed state compared to administration in the fasted state (Treatment I), when measured by C_max_ or AUC_0–t_ (approximately 36% higher; *p* = 0.026 and 26% higher; *p* = 0.024, respectively).

There was no statistically significant difference in total exposure, when measured by AUC_0–t_ or in peak exposure measured by C_max_ for Treatment J compared to Treatment H when both were administered in the fed state (*p* = 0.53 and *p* = 0.33, respectively).

## Discussion

GDC-0810 was provided for use in Phase I/II clinical studies in a tablet formulation using NMG salt with 10% SLS and 15% NaHCO_3_. However, high drug loading and atypical levels of SLS presented challenges for drug product manufacturability. The formulation showed poor powder flow properties, which resulted in high variability of tablet weight and tablet hardness. In addition, sufficient tablet hardness suitable for downstream coating and handling could not be achieved with this formulation. Hence, further formulation optimization was required to provide a tablet product for use in future clinical studies or for commercial use.

The strategy for Phase III formulation development was focused on the optimization of the levels of excipients in Phase II tablet formulation to improve manufacturability without decreasing drug exposure and stability. A series of design of experiments (DOE) were conducted with the aim of evaluating the impact of formulation components on tablet disintegration, *in vitro* dissolution, and drug product manufacturability. A significant reduction in the tablet hardness and prolonged tablet disintegration time were observed with increasing the level of SLS in the formulation (data not shown). The results also revealed that the level of sodium bicarbonate impacted *in vitro* dissolution of GDC-0810 the most. The dissolution rate increased with increasing levels of sodium bicarbonate in the formulation (7). To confirm the effect of these excipients on *in vivo* drug exposure and identify the optimal levels of excipients in Phase III formulation, a relative bioavailability study was planned.

Traditional approaches to assess dissolution characteristics of the formulation include *in vitro* dissolution methods followed by preclinical studies. The poor correlation between human and animal bioavailability posed a risk for the optimized formulation that necessitated its clinical testing (15,16). Nonetheless, conventional clinical assessment was time-consuming and costly because it was based on multiple cycles of *in vitro* and preclinical testing for formulation selection, scaling up, and generating stability data prior to clinical dosing. To evaluate PK performance of the formulation efficiently, RapidFACT® was employed to accelerate the screening of the new formulations in human subjects, by avoiding the need to pre-determine and pre-manufacture clinical formulation compositions based on such surrogate measures. In addition, a two-dimensional formulation design space was used within the RapidFACT® program to maximize the freedom to adjust SLS and NaHCO_3_ levels in the formulation in response to emerging PK data.

This “Make and Test in Parallel” strategy therefore allowed the rapid and iterative adjustment of formulation compositions in real-time to maximize the potential to achieve the TPP within a single clinical study. RapidFACT® has been previously demonstrated to successfully optimize dissolution characteristics of the formulation. One example of successful implementation of RapidFACT® is the prostate cancer drug abiraterone acetate (Zytiga), which had very low bioavailability and a significant positive food effect in men due to inadequate and variable dissolution. The manufacturer then developed a novel formulation with improved solubility and dissolution characteristics. Using RapidFACT®, three consecutive reduced doses were tested in the presence of food. A 75% dose reduction with significant reduction of inter-individual variability was achieved with the novel formulation in a single clinical PK trial (17). RapidFACT® also allowed the potential for post study analysis of *in vitro* and *in vivo* datasets to increase product and process understanding in early development. Although not an objective of the current study previous publications have demonstrated for example how RapidFACT® has been used to establish an *in vitro-in-vivo* correlation (18).

In the current study, the level of SLS in the range of 0–4% and the level of NaHCO_3_ in the range of 5–15% were defined as the two-dimensional formulation design space. The results of formulation development DOE showed that Phase III Prototype 1 tablet formulation containing 2% SLS and 15% NaHCO_3_ demonstrated the best performance in terms of manufacturability and *in vitro* drug release, followed by Phase III Prototype 2 tablet formulation containing 2% SLS and 10% NaHCO_3_. Therefore, these two formulations were first selected to be evaluated in Part 1 as compared to the reference Phase I/II tablet formulation. The human PK data of dosing periods 1–3 showed that the plasma GDC-0810 exposure with Phase III Prototype 1 formulation (Treatment B) offered maximum exposure. The reduction in SLS content from 10 to 2% resulted in a slightly lower C_max_ (statistically non-significant) while maintaining the extent of exposure (AUC_0-t_) of GDC-0810 (GMR 90%), suggesting that 2% SLS in the formulation would be sufficient to deliver the comparable exposure of GDC-0810 as 10% SLS. Phase III Prototype 2 formulation (Treatment C) showed slightly lower C_max_ but comparable AUC_0-t_ as the Prototype 1 formulation, suggesting that the level of NaHCO_3_ varying from 15 to 10% did not significantly affect the *in vivo* GDC-0810 exposure. Therefore, an interim decision was made, based on the available safety, tolerability, and PK data following completion of Period 3, to select the Phase III Prototype 3 formulation containing 5% NaHCO_3_ to be manufactured and dosed in Period 4 in Part 1. It appears that C_max_ and AUC_0-t_ of the Prototype 3 formulation was significantly lowered. These results suggest that at least 15% NaHCO_3_ is needed to maintain the plasma exposure of GDC-0810 comparable to that of the Phase I/II formulation. Based on these findings from Part 1 using the ‘Make and Test in Parallel’ approach, the composition of excipients was set to 2% SLS and 15% NaHCO_3_ for further testing in Part 2.

In Part 2 of the study, GDC-0810 PK with the free acid formulation (Treatment F) and the spray-dried amorphous solid dispersion formulation (Treatment G) were assessed and compared with Phase II NMG salt formulation (Treatment E). The test formulations, F and G, included SLS content of 2% and NaHCO_3_ content of 15% and were administered following a low-fat meal. There was no statistically significant difference between the formulations tested, suggesting that the form of active pharmaceutical ingredient in these formulations of GDC-0810 did not influence the bioavailability of the drug. Moreover, the initial plan to develop the free acid form for Phase III formulation was impacted by the emergence of a new polymorph with 3-folds reduction in solubility. Therefore, based on these findings as well as the results of Part 1 and Part 2, Phase III Prototype 1 formulation containing NMG salt with 2% SLS and 15% NaHCO_3_ was selected for further testing in Part 3.

In Part 3 of the study, the Phase III Prototype 1 formulation, containing NMG salt, 2% SLS and 15% NaHCO_3_, was administered following a low-fat meal but at a higher dose of 600 mg GDC-0810. The plasma exposure of GDC-0810 with Phase III prototype 1 formulation was comparable with the Phase I/II formulation even at the clinically relevant dose of 600 mg, confirming the findings at 200 mg in Part 1. These results are in line with the expected PK linearity of GDC-0810 with respect to dose.

In addition, when the PK of Phase III prototype formulation 1 at 600 mg dose in fed state (low-fat) was compared with fasted state in Part 3, approximately 30% increase in bioavailability with food was observed. The increase in exposure in the presence of food was statistically significant and could be a result of food aiding the solubility of GDC-0810. Furthermore, the t_max_ in the fed state was increased and is expected to be a result of a slower rate of gastric emptying observed in the fed state.

Variability in exposure parameters of GDC-0810 was extensive in this study, with geometric CV% ranging between 26.2 and 85.8%. This is not unusual for compounds requiring excipients to increase solubility. The elimination half-life was unchanged for all formulations administered at all dose levels tested, with values of between 13.4 h and 18.6 h.

## Conclusions

Overall, the study successfully identified a Phase III formulation with a reduced SLS content which, when administered after a low-fat meal, gave comparable exposure to the Phase II formulation. Thus, our novel ‘Make and Test in Parallel’ approach allowed the optimization of a GDC-0810 formulation in the most time- and cost-efficient fashion based on clinical data. In addition, given the higher exposure with food, the administration of GDC-0810 was recommended with food.

## Abbreviations

AE: Adverse event
ANOVA: Analysis of variance
API: Active Pharmaceutical Ingredient
AUC_0-inf_: Area under the curve (zero to infinity)
AUC_0-t_: Area under the curve (from time to last measurable reading)
CI: Confidence intervals
C_max_: Maximum drug concentration in plasma
CMC: Chemistry, Manufacturing, and Controls
CTCAE: Common terminology criteria for adverse events
CV: Coefficient of variance
DOE: Design of experiments
ECG: Electrocardiogram
ER: Estrogen Receptor
ER^+^: Estrogen Receptor positive
GCP: Good Clinical Practices
GMP: General Manufacturing procedures
GMR: Geometric mean ratio
HER: Human epidermal growth factor negative
ICH E6: International Conference on Harmonisation of Technical Requirements for Pharmaceuticals for Human Use
LLOQ: Lowe limit of quantification
NaHCO_3_: Sodium Bicarbonate
NMG: N-Methyl-D-glucamine
PK: Pharmacokinetics
RapidFACT®: Rapid Formulation development and Clinical Testing
SAE: Severe adverse event
SLS: Sodium Lauryl Sulfate
TEAE: Treatment emergent adverse effects
TPP: Target product profile
